# Supplementary material for: Neoantigen-specific CD8 T cell responses in the peripheral blood following PD-L1 blockade might predict therapy outcome in metastatic urothelial carcinoma
Source: Nat Commun. 2022 Apr 11;13:1935. doi: 10.1038/s41467-022-29342-0 (PMC9001725; doi:10.1038/s41467-022-29342-0)
Supplement: Supplementary file 1 — Supplementary Information [file 41467_2022_29342_MOESM1_ESM.pdf]

Supplementary Information

Neoantigen-specific CD8 T cell responses in the peripheral blood following PD-L1 blockade might predict therapy outcome in metastatic urothelial carcinoma

Jeppe Sejerø Holm[1*], Samuel A. Funt[2,3*], Annie Borch[1], Kamilla Kjærgaard Munk[1], Anne-Mette Bjerregaard[1], James L. Reading[5], Colleen Maher[2,3,7], Ashley Regazzi[2,3], Phillip Wong[2,3,7], Hikmat Al-Ahmadie[6], Gopa Iyer[2,3], Tripti Tamhane[1], Amalie Kai Bentzen[1], Nana Overgaard Herschend[1], Susan De Wolf[2], Alexandra Snyder[2,3], Taha Merghoub[2,3,7], Jedd D. Wolchok[2,3,7,8], Morten Nielsen[4], Jonathan E. Rosenberg[2,3], Dean F. Bajorin[2,3*], Sine Reker Hadrup[1*§]

Supplementary Figures 1-6

Supplementary Tables 1-3

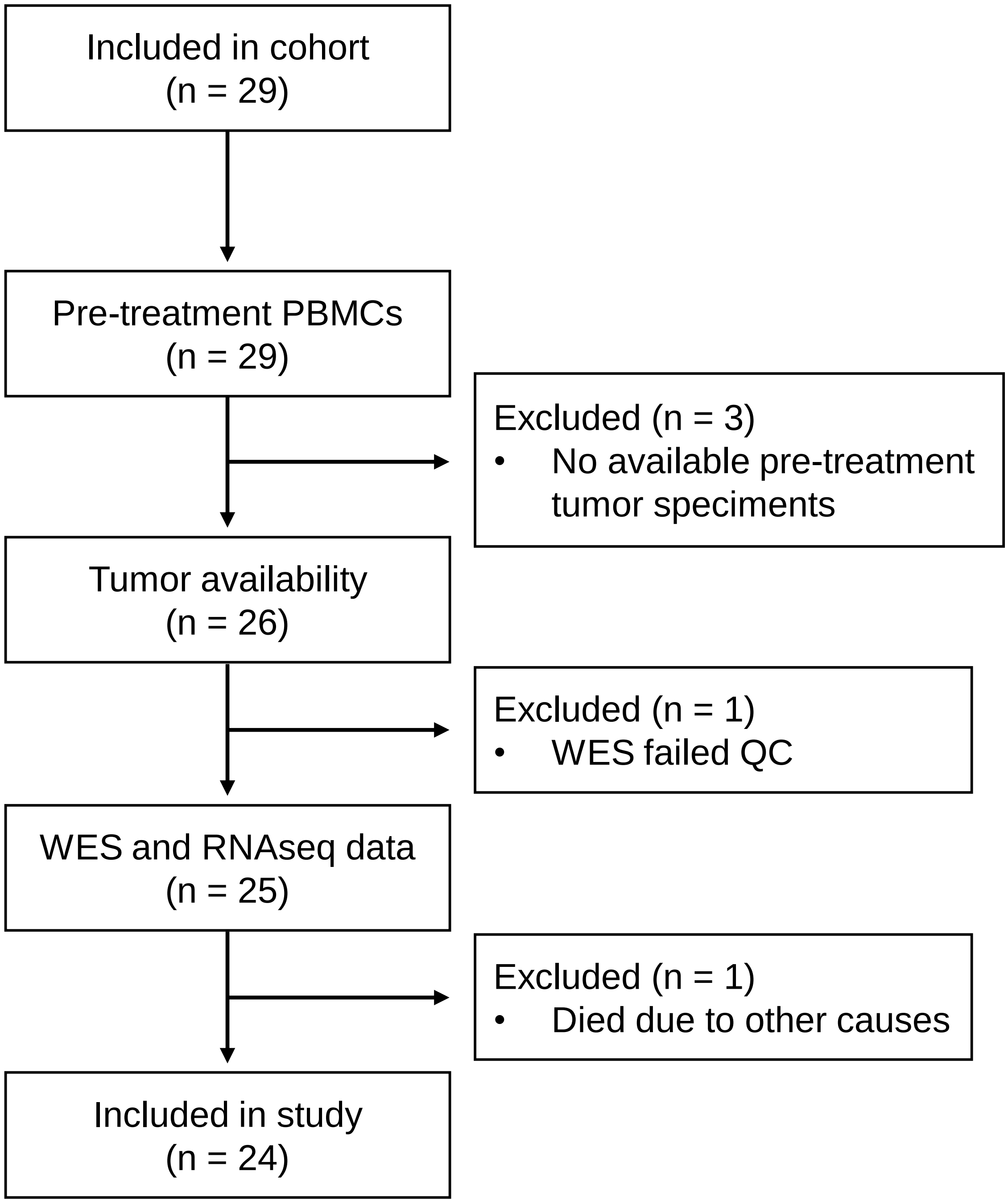

Supplementary Figure 1

Supplementary Fig. 1 Consort diagram of patients in cohort included in previous study (Snyder et al., 2017) and inclusion criteria for patient samples analyzed here. Patients #0979, #7592, and #8214 had no available pre-treatment archival tumor speciments, QC for WES of patient #4072 failed, and patient #9881 died of other causes. These patients were excluded from this study.

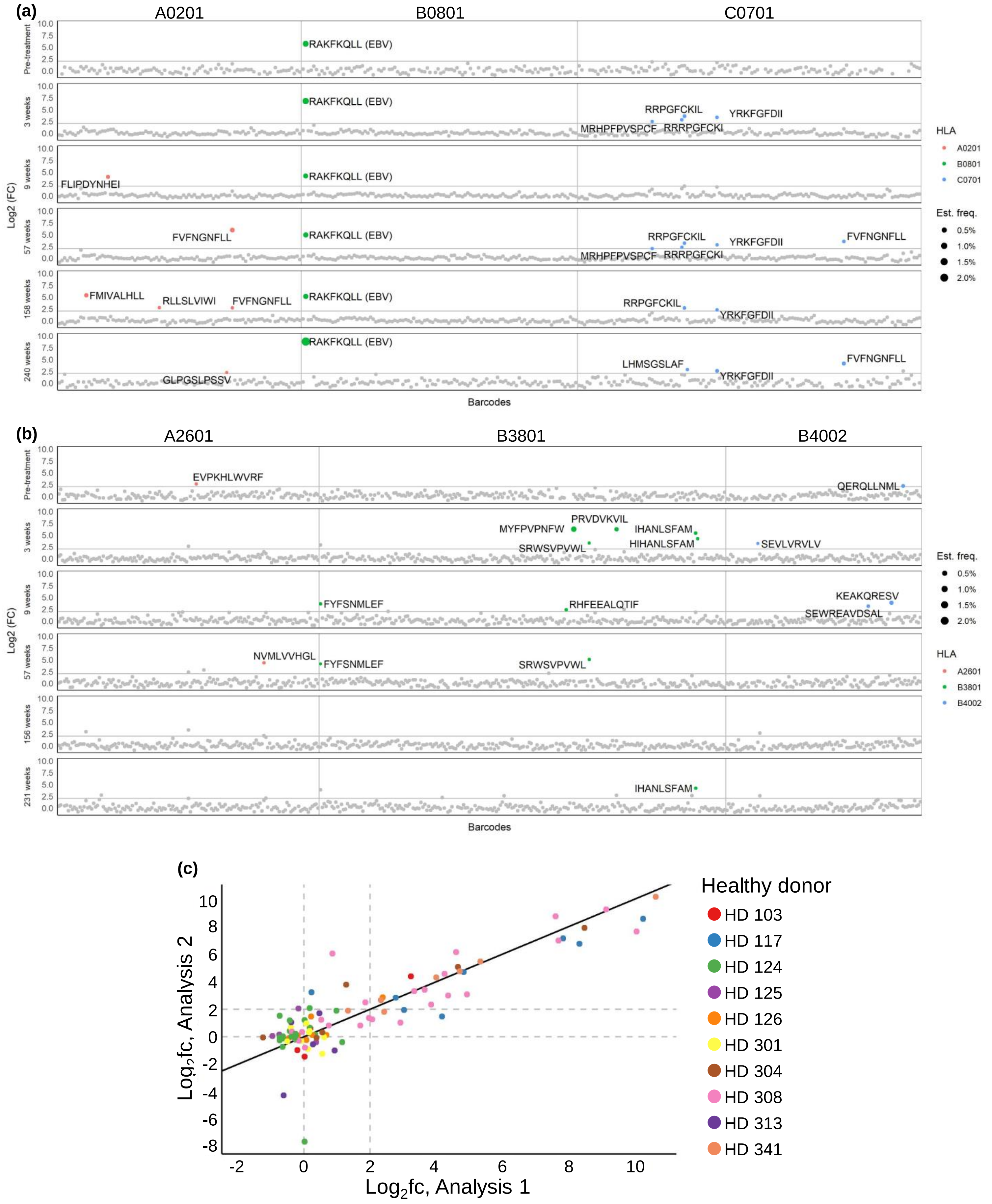

Supplementary Figure 2

Supplementary Fig. 2 Detection of neoepitope-reactive T cell responses in mUC cancer patients. Additional representative output from patients **(a)** #2131 and **(b)** #5037, screening of detected NART responses. Log_2FC of sequenced pMHC associated barcodes enriched by T cell sorting over the input library at stated timepoints. Labelled points; Log_2 FC > 2, count fraction > 0.1 % and $p < 0.001$, determined as T cell responses. Colored based on peptide-presenting HLA-type, text labelled with peptide sequence, and sized according the estimated frequency of the peptide-recognizing T cell population. For virus-derived peptides the virus is stated in brackets. Grey points represent non-enriched barcodes. Horizontal line at Log_2 fc = 2. Vertical line separating peptide-presenting HLA-types. **(c)** Log_2FC of viral CMV, EBV, and FLU-derived pMHC-associated barcodes (n = 72) from duplicate stainings of HD PBMC controls (n = 10). Source data are provided as a Source Data file.

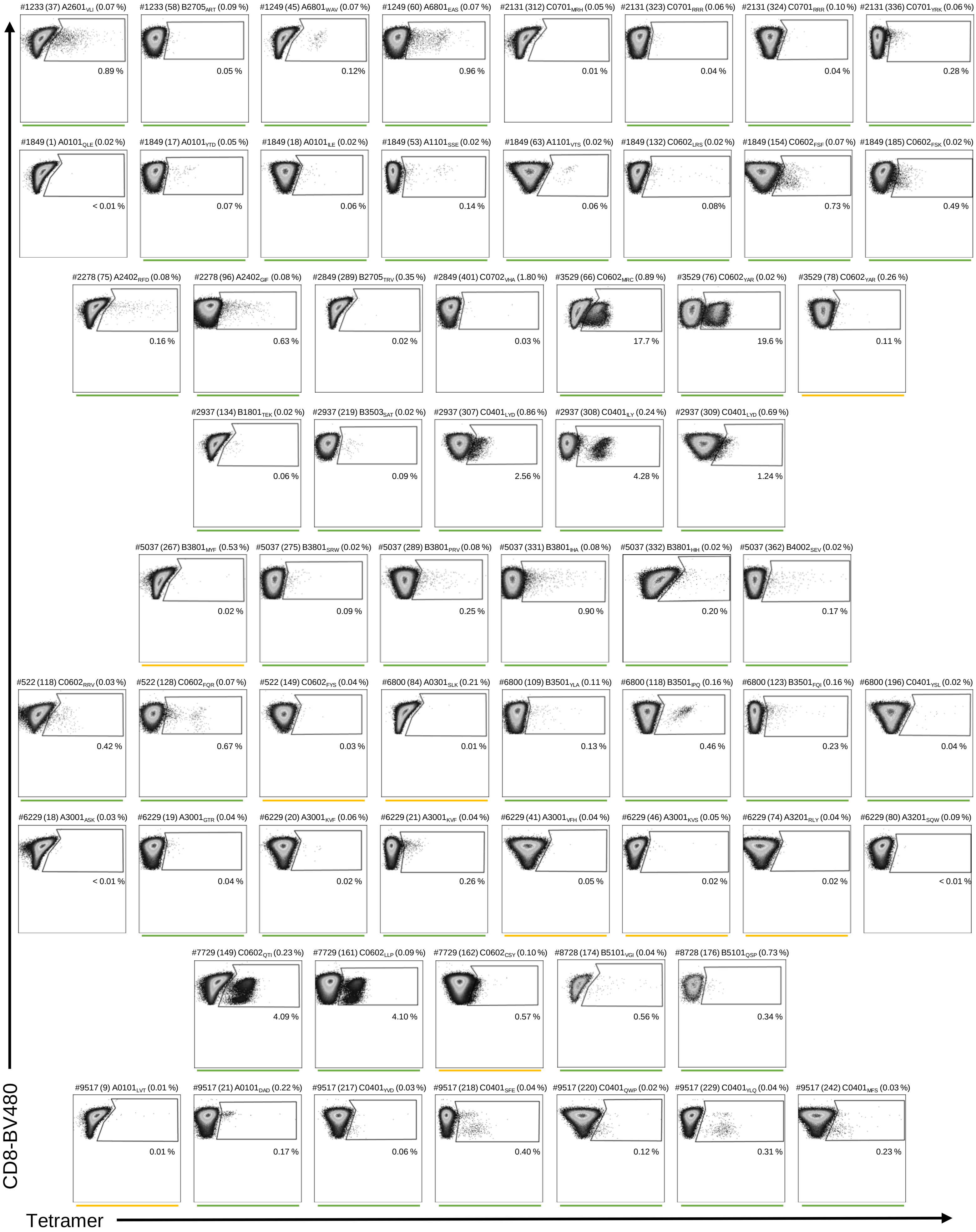

Supplementary Figure 3

Supplementary Fig. 3 Tetramer pMHC stainings for NART response validations. CD8+ cells shown, gated for tetramer+ populations. Selected patient PBMC samples, either at 3- or 9-weeks post-treatment depending on sample availabily at time of study, were stained with tetramers generated for recognized neoepitopes seen in multimer screening at given timepoint. Neoepitope number and sequence, presenting HLA-type, estimated frequency from multimer screening, and tetramer+ NART frequency listed for each pMHC. Green markings indicate positive validation of NART response, with yellow markings indicating NART responses at border of detection level using tetramer stainings. PBMCs from 3-weeks post-treatment; patients #1249, #2131, #2278, #2849, #2937, #3529, #5037, #522, #6229, #6800, #7729, #8728, and #9517. PBMCs from 9-week post-treatment; patients #1233 and #1849.

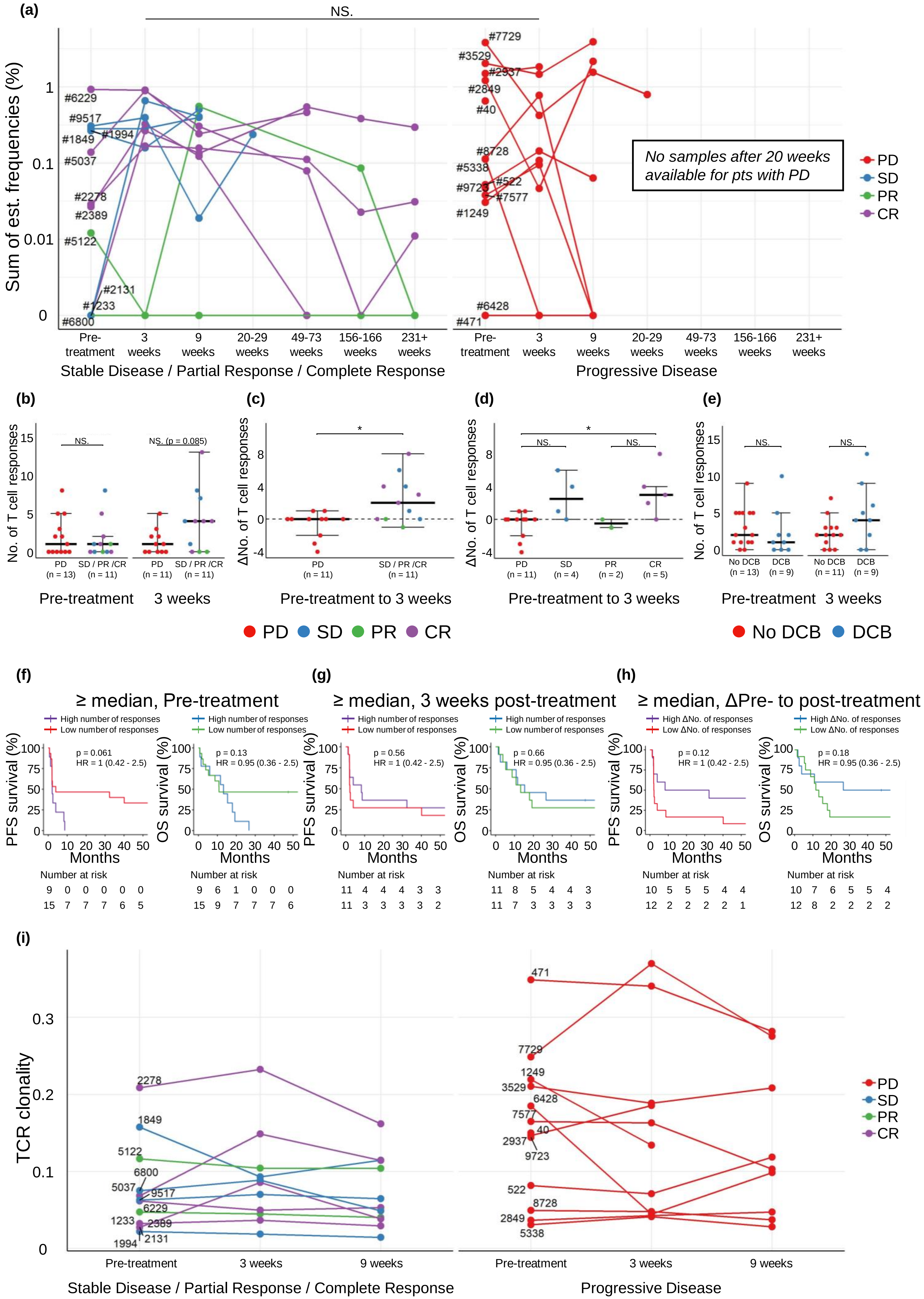

Supplementary Figure 4

Supplementary Fig. 4 Impact of NARTs in anti-PD-L1 treatment. **(a)** SEFs for NART populations over course of therapy. Points at respective time point indicate screening of the given sample. Patients clustered according to patients with SD, PR, and CR (n = 11 patients) and with PD (n = 13 patients). **(b)** Number of NART responses for patients and **(c-d)** the change from pre- to 3-week post-treatment, between SD/PR/CR and PD patients, only for NART responses towards neopeptides with EL%Rank < 0.5 and expression level > 2 TPM. **(e)** Number of NART responses for patients with or without DCB. **(f-h)** Kaplan-Meier estimation curves for patient PFS and OS grouped according to High or Low number of NART responses at **(f)** pre-treatment (> median = 2 responses) or at **(g)** 3-week post-treatment (≥ median = 3 responses), or **(h)** change in number of NART responses (> median = 0 response increase). **(i)** Bulk PBMC TCRb cloanlity at pre-treatment, 3 weeks- and 9 weeks post-treatment. Points at respective time point indicate PBMC sample and TCRb sequencing data availability. For b)-e) groups were compared using non-parametric two-sided Mann-Whitney test and Kruskal-Wallis Dunn's multiple comparison test for d). For a)-e), data is presented as median values +/- largest/smallest value within upper/lower quartile +/- 1.5 IQR. NS.: Not Significant, *: $p < 0.05$. Source data are provided as a Source Data file.

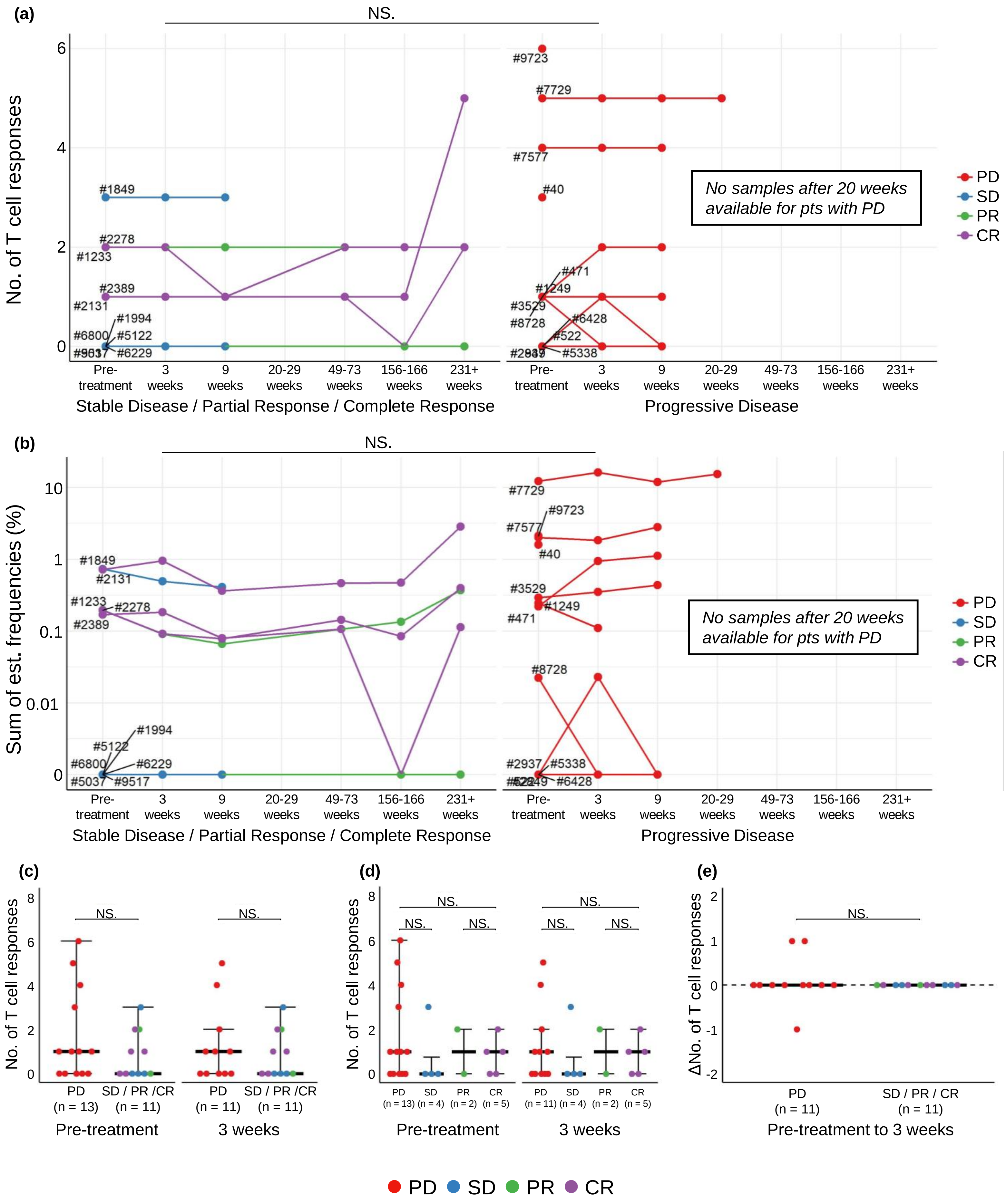

Supplementary Figure 5

Supplementary Fig. 5 VARTs in anti-PD-L1 treatment. Timeline of **(a)** number of VART responses and **(b)** SEF for VART populations over course of therapy. Points at respective time point indicate screening of the given sample. Patients clustered according to patients with SD, PR, and CR (n = 11 patients) and with PD (n = 13 patients). **(c)** Number of VART responses for patients, clustered according to disease control and **(d)** according to RECIST criteria, at pre- and 3-week post-treatment. **(e)** The change in number of VART responses for patients, clustered according to disease control. For c) + e) groups were compared using non-parametric two-sided Mann-Whitney test and Kruskal-Wallis Dunn's multiple comparison test for d). For c)-e), data is presented as median values +/- largest/smallest value within upper/lower quartile +/- 1.5 IQR. NS.: Not Significant. Source data are provided as a Source Data file.

Supplementary Figure 6

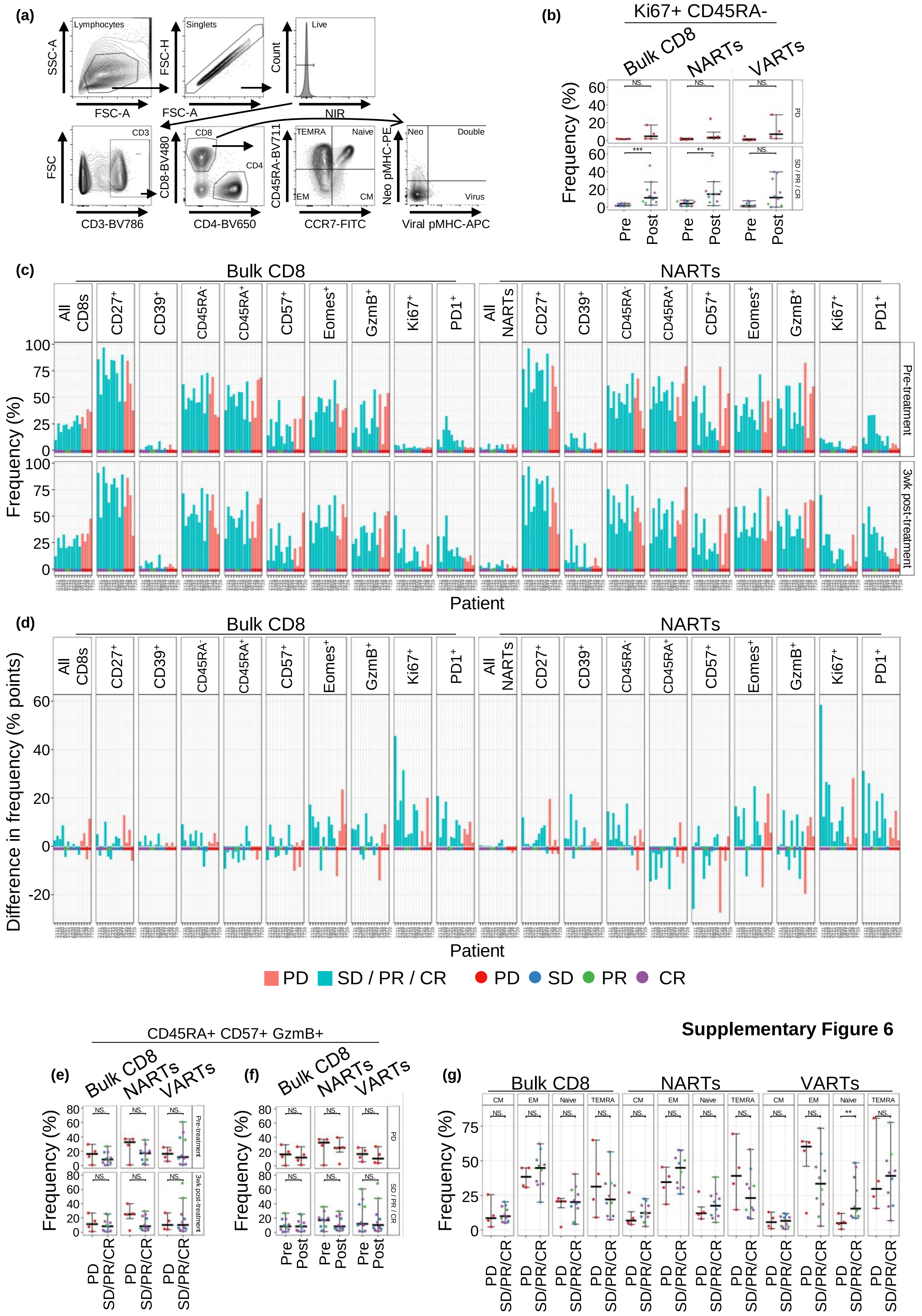

Supplementary Fig. 6 Phenotypic characterisation of bulk CD8 T cells, NARTs and VARTs. **(a)** Gating strategy for sorting and characterisation of PE-neo- and APC-viral pMHC multimer-binding singlet, Live (NIR-), CD3+, CD8+/CD4- lymphocytes. **(b)** Parent population frequencies of Ki67+CD45RA- bulk CD8 T cells, NARTs, and VARTs for SD/PR/CR (n = 10 patients) and PD patients (n = 4 patients) at pre- and 3 weeks post-treatment. **(c-d)** Parent population frequencies of bulk CD8 T cells and NARTs for all non-lineage parameters at pre- and 3 weeks post-treatment, and the change in frequency between time points. **(e-f)** Parent population frequencies of triple-positive CD45RA+ CD57+ GzmB+ bulk CD8 T cells, NARTs, and VARTs for PD (n = 4 patients) and SD/PR/CR patients (n = 10 patients) at pre- and 3 weeks post-treatment. Mann-Whitney test for l)-p). **(g)** Parent population frequencies of Central- (CM) and Effector Memory (EM), Naive and TEMRA bulk CD8 T cells, NARTs, and VARTs for PD (n = 4 patients) and SD/PR/CR patients (n = 10 patients) at 3 weeks post-treatment. For b) + e)-g) groups were compared using non-parametric two-sided Mann-Whitney test and data is presented as median values +/- largest/smallest value within upper/lower quartile +/- 1.5 IQR NS.: Not Significant, *: $p < 0.05$, **: $p < 0.01$, ***: $p < 0.001$. wk: week, Pre: Pre-treatment, Post: 3 weeks post-treatment. Source data are provided as a Source Data file.

Pt #	T cell neoepitope responses, per time point						
	Pre-treatment	3 weeks	9 weeks	20-29 weeks	49-73 weeks	156-166 weeks	231+ weeks
**#40**	#128 HSSEVTMTL (A3201)<br>#138 TLIFARLTI (A3201)<br>#388 SYDTALQARI (C0401)<br>#414 VFMEENSKL (C0401)<br>#463 IRLRPRHVL (C0701)	*Subsequent samples not available*					
**#9723**	#96 QQQQQAQTL (B0801)	*Subsequent samples not available*					
**#1249**	-<br>#60 EASVKVIHR (A6801)	#45 WAVFPSIVGR (A6801)<br>#60 EASVKVIHR (A6801)	*Subsequent samples not available*				
**#2937**	#42 ATIKVNGTI (A3201)<br>#60 SLLEHVEEY (A3201)<br>#91 MVEAGAGTSW (B1801)<br>#95 SEFDNLRTL (B1801)<br>-<br>#194 EPWVSIKKF (B3503)<br>-<br>#234 FPLPWWDLM (B3503)<br>#307 LYDQGYTSL (C0401)<br>#308 ILYDQGYTSL (C0401)<br>#309 LYDQGYTSLG (C0401)	-<br>-<br>-<br>-<br>#134 TEKPPYIEV (B1801)<br>-<br>#219 SATEAFGEL (B3503)<br>-<br>#307 LYDQGYTSL (C0401)<br>#308 ILYDQGYTSL (C0401)<br>#309 LYDQGYTSLG (C0401)	*Subsequent samples not available*				
**#1994**	-<br>-<br>#89 YTYAAMLRI (C0702)<br>-<br>#147 LLLAAVVSH (A0301)<br>-<br>#186 NSVPPPPPL (B0702)	-<br>#23 KPMTLFQIQF (B0702)<br>-<br>-<br>#147 LLLAAVVSH (A0301)<br>-<br>-	#21 RPRLLLLVL (B0702)<br>-<br>-<br>#115 FYILSSGLI (C0702)<br>-<br>#164 GLPNVFGLGR (A0301)<br>-	*Subsequent samples not available*			
**#522**	#19 SFLVHPYGF (A2402)<br>-<br>#128 FQRFHKLHYL (C0602)<br>-	-<br>#118 RRVAMRRWI (C0602)<br>#128 FQRFHKLHYL (C0602)<br>#149 FYSSRKRLL (C0602)	-<br>#118 RRVAMRRWI (C0602)<br>#128 FQRFHKLHYL (C0602)<br>-	*Subsequent samples not available*			
**#6428**	No responses	No responses	No responses	*Subsequent samples not available*			
**#7577**	-<br>#9 CMDFNSNGKY (A0101)	#1 ANDNSPFMLY (A0101)<br>#9 CMDFNSNGKY (A0101)	No responses	*Subsequent samples not available*			
**#8728**	#118 TEKPIQRNPG (B4403)<br>#141 SPLPQSPQV (B5101)<br>#144 LPLKLQSEV (B5101)<br>#153 HPQDFRDHPV (B5101)<br>#170 QLPLKLQSEV (B5101)<br>-<br>-	-<br>-<br>-<br>-<br>-<br>#174 VGIFILCTV (B5101)<br>#176 QSPQVLQQL (B5101)	No responses	*Subsequent samples not available*			
**#471**	No responses	No responses	No responses	*Subsequent samples not available*			
**#3529**	#66 MRCLVQHIL (C0602)<br>#76 YARFLQSNAY (C0602)<br>#78 YRAQVYVPV (C0602)<br>-<br>#149 KGYEGYYVL (C0602)<br>#177 YRSGHQLHC (C0602)<br>-	#66 MRCLVQHIL (C0602)<br>#76 YARFLQSNAY (C0602)<br>#78 YRAQVYVPV (C0602)<br>-<br>-<br>-<br>-	#66 MRCLVQHIL (C0602)<br>#76 YARFLQSNAY (C0602)<br>#78 YRAQVYVPV (C0602)<br>#130 RTFSMQVAL (A0205)<br>#149 KGYEGYYVL (C0602)<br>#177 YRSGHQLHC (C0602)<br>#179 AFQIAMKLL (A2402)	*Subsequent samples not available*			
**#5338**	#161 HPHPHPHAF (C0304)	No responses	No responses	*Subsequent samples not available*			
**#2849**	#249 LPEARRPRL (B0702)<br>-<br>#298 GRHILVAWK (B2705)<br>#371 YFSQEQWGL (C0702)<br>#385 YFKSDELQF (C0702)<br>#401 VHARVINFF (C0702)	-<br>-<br>-<br>-<br>-<br>#401 VHARVINFF (C0702)	-<br>#289 TRVKCVVSM (B2705)<br>-<br>-<br>-<br>#401 VHARVINFF (C0702)	*Subsequent samples not available*			
**#7729**	-<br>#149 QTIDKAKYI (C0602)<br>#150 SQRETTWTF (C0602)<br>#161 LLPPYKQSI (C0602)<br>#162 CSYSEPHYM (C0602)<br>#198 LLPPYKQSI (C0701)	-<br>#149 QTIDKAKYI (C0602)<br>-<br>#161 LLPPYKQSI (C0602)<br>#162 CSYSEPHYM (C0602)<br>-	#132 IRTDSVLIL (C0602)<br>#149 QTIDKAKYI (C0602)<br>#150 SQRETTWTF (C0602)<br>#161 LLPPYKQSI (C0602)<br>#162 CSYSEPHYM (C0602)<br>-	-<br>#149 QTIDKAKYI (C0602)<br>-<br>#161 LLPPYKQSI (C0602)<br>#162 CSYSEPHYM (C0602)<br>-	*Subsequent samples not available*		
**#9517**	-<br>-<br>#21 DADLLRPHAY (A0101)<br>#212 TRLFLFHLL (B3801)<br>-<br>-<br>-<br>-<br>-	-<br>#9 LVTEDTTICY (A0101)<br>#21 DADLLRPHAY (A0101)<br>-<br>#217 YVDYPIYDML (C0401)<br>#218 SFEEYLKLL (C0401)<br>#220 QWPPFVVTL (C0401)<br>#229 YLQTYGAEL (C0401)<br>#242 MFSGVAVYL (C0401)	-<br>#9 LVTEDTTICY (A0101)<br>-<br>-<br>-<br>-<br>-<br>-<br>-	#8 VTEDTTICY (A0101)<br>#9 LVTEDTTICY (A0101)<br>#21 DADLLRPHAY (A0101)<br>-<br>-<br>-<br>-<br>#229 YLQTYGAEL (C0401)<br>-	*Subsequent samples not available*		

Supplementary Table 1

Pt #	T cell neoepitope responses, per time point						
	Pre-treatment	3 weeks	9 weeks	20-29 weeks	49-73 weeks	156-166 weeks	231+ weeks
	#1 QLEQLMQLY (A0101)	-	#1 QLEQLMQLY (A0101)				
	#17 YTDQISKYA (A0101)	#17 YTDQISKYA (A0101)	#17 YTDQISKYA (A0101)				
	-	-	#18 ILEYTDQISKY (A0101)				
	-	-	#53 SSEPPFGPK (A1101)				
	#63 VTSEVSNLK (A1101)	-	#63 VTSEVSNLK (A1101)				
	#132 LRSRHSTRI (C0602)	#132 LRSRHSTRI (C0602)	#132 LRSRHSTRI (C0602)				
	-	#133 LRACTRSSM (C0602)	-				
**#1849**	#146 LRSSQRMVI (C0602)	#146 LRSSQRMVI (C0602)	#146 LRSSQRMVI (C0602)		*Subsequent samples not available*		
	-	#150 FRNSANATSL (C0602)	#150 FRNSANATSL (C0602)				
	#152 SRYGGGLAV (C0602)	#152 SRYGGGLAV (C0602)	#152 SRYGGGLAV (C0602)				
	#154 FSFSKSRRI (C0602)	#154 FSFSKSRRI (C0602)	#154 FSFSKSRRI (C0602)				
	#155 FNYKPLHTI (C0602)	#155 FNYKPLHTI (C0602)	#155 FNYKPLHTI (C0602)				
	#164 LRSVSPWTY (C0602)	-	#164 LRSVSPWTY (C0602)				
	-	-	#167 MSMPKGRVV (C0602)				
	#185 FSKSRRILL (C0602)	#185 FSKSRRILL (C0602)	#185 FSKSRRILL (C0602)				
		#84 SLKRLRNTK (A0301)	-				
		#109 YLAKHTILY (B3501)	-				
**#6800**	No responses	#118 IPQDSIAQIY (B3501)	-		*Subsequent samples not available*		
		#123 FQISSGISF (B3501)	-				
		-	#190 KYMQMNYAL (C0401)				
		#196 YSLELGMTL (C0401)	-				
	#14 RMHYLPQLK (A3001)	-	-		-		
	-	#18 ASKPIFMDR (A3001)	-		-		
	-	#19 GTRMPTSTY (A3001)	-		-		
	-	#20 KVFSHHAYI (A3001)	-		-		
	-	#21 KVFSHHAYF (A3001)	-		-		
	#36 HQRPGHLLA (A3001)	-	-		-		
	-	#41 VFHTPTVIK (A3001)	-		-		
	-	#46 KVSFSGCMVK (A3001)	-		-		
	-	#74 RLYTGKKPY (A3201)	-		-		
**#6229**	-	#80 SQWRKAPGW (A3201)	-	*Sample not available*	-	*Subsequent samples not available*	
	-	-	#94 IYHQPTHLW (A3201)		-		
	-	#201 FQLDQITAL (C0602)	-		-		
	#205 IYHQPTHLW (C0602)	#205 IYHQPTHLW (C0602)	-		-		
	-	#226 RHRDPVPEL (C0602)	-		-		
	-	-	-		#230 FRSRWKEQY (C0602)		
	-	#234 SRWNMSRRL (C0602)	-		-		
	#235 SRPLPVAAV (C0602)	-	-		-		
	#243 WRSERRSWV (C0602)	#243 WRSERRSWV (C0602)	#243 WRSERRSWV (C0602)		#243 WRSERRSWV (C0602)		
	-	-	#250 SRRVREASL (C0602)		-		
	#41 EVPKHLWVRF (A2601)	-	-		-		
	-	-	-		#73 NVMLVVHGL (A2601)		
	-	-	#136 FYFSNMLEF (B3801)		-		
	-	-	#263 RHFEEALQTIF (B3801)		-		
	-	-	-		-		
	-	#267 MYFPVPNFW (B3801)	-		-		
**#5037**	-	#275 SRWSVPVWL (B3801)	-	*Sample not available*	#275 SRWSVPVWL (B3801)	No responses	No responses
	-	#289 PRVDVKVIL (B3801)	-		-		
	-	#331 IHANLSFAM (B3801)	-		-		
	-	#332 HIHANLSFAM (B3801)	-		-		
	-	#362 SEVLVRVLV (B4002)	-		-		
	-	-	#419 SEWREAVDSAL (B4002)		-		
	-	-	#431 KEAKQRESV (B4002)		-		
	#437 QERQLLNML (B4002)	-	-		-		
	#1 CIDFQPDIY (A0101)	#1 CIDFQPDIY (A0101)	#1 CIDFQPDIY (A0101)				-
	-	#2 SCIDFQPDIY (A0101)	#2 SCIDFQPDIY (A0101)				-
**#2389**	-	-	-	*Sample not available*	No responses	No responses	#26 KWPECEKVF (A2402)
	-	#141 TELGTAAKL (B4001)	#141 TELGTAAKL (B4001)				-
	#159 NEASLSFQAL (B4001)	#159 NEASLSFQAL (B4001)	-				-
		-	-		-	#15 FMIVALHLL (A0201)	-
		-	#23 FLIPDYNHEI (A0201)		-	-	-
		-	-		-	#40 RLLSLVIWI (A0201)	-
		-	-		-	-	#63 GLPGSLPSSV (A0201)
		-	-		#65 FVFNGNFLL (A0201)	#65 FVFNGNFLL (A0201)	-
**#2131**	No responses	#312 MRHPFPVSPCF (C0701)	-	*Sample not available*	#312 MRHPFPVSPCF (C0701)	-	-
		#323 RRRPGFCKI (C0701)	-		#323 RRRPGFCKI (C0701)	-	-
		#324 RRPGFCKIL (C0701)	-		#324 RRPGFCKIL (C0701)	#324 RRPGFCKIL (C0701)	-
		-	-		-	-	#325 LHMSGSLAF (C0701)
		#336 YRKFGFDII (C0701)	-		#336 YRKFGFDII (C0701)	#336 YRKFGFDII (C0701)	#336 YRKFGFDII (C0701)
		-	-		#383 FVFNGNFLL (C0701)	-	#383 FVFNGNFLL (C0701)
	-	-	-		-	#13 LLAKGLVLL (A0201)	-
	-	-	-		-	-	#44 RFDAFVLFL (A0201)
	-	-	-		#71 RWYAICHPFMF (A2402)	-	-
**#2278**	-	-	-	*Sample not available*	#72 WYAICHPFMF (A2402)	-	-
	#75 RFDAFVLFL (A2402)	#75 RFDAFVLFL (A2402)	-		#75 RFDAFVLFL (A2402)	-	-
	-	#96 GIFGLNLALF (A2402)	-		-	-	-
	-	-	#118 FYRALMSNTY (A2402)		-	-	-
			#37 VLIFAVVGM (A2601)			-	
**#1233**	No responses	No responses	-	*Samples not available*		#56 EDFLLHINF (A2601)	No responses
			#58 ARTVRPVSL (B2705)			-	
**#5122**	#154 GRGEGPIWL (B3801)	No responses	No responses	*Samples not available*		No responses	No responses

Supplementary Table 1 cont.

Supplementary Table 1 Detailed patient T cell neoepitope response data. The patient-specific peptide no., peptide sequence, and peptide-presenting HLA is stated for each T cell neoepitope response. '-' or 'No responses' indicate no neoepitope T cell responses were detected towards the specific neoepitope or at all in the PBMC sample at the given timepoint. Source data are provided as a Source Data file.

Patient	Clinical characteristics				
	DCB *(PFS > 6 months)*	Outcome *(Best RECIST 1.1)*	PFS *(Days)*	OS *(Days)*	Ultimate time point *(Weeks)*
**#40**	No DCB	PD[1]	20	24	0
**#9723**	No DCB	PD[1]	19	22	0
**#1249**	No DCB	PD[1]	41	54	3
**#2937**	No DCB	PD	37	44	3
**#1994**	No DCB	SD	121	403	9
**#522**	No DCB	PD	58	76	9
**#6428**	No DCB	PD	61	163	9
**#7577**	No DCB	PD	60	117	9
**#8728**	No DCB	PD	61	546	9
**#471**	No DCB	PD	61	329	9
**#3529**	No DCB	PD	64	182	9.4
**#5338**	No DCB	PD	67	266	9.7
**#2849**	No DCB	PD	75	586	11
**#7729**	No DCB	PD	60	354	20
**#9517**	No DCB	SD	121	331	29
**#1849**	DCB	SD	265	468	9
**#6800**	DCB	SD	985	1445	9
**#6229**	DCB	CR	252	812	49.3
**#5037**	DCB	CR	1954	1954	231.1
**#2389**	DCB	CR	1990	1990	237.1
**#2131**	DCB	CR	1965	1965	240
**#2278**	DCB	CR	2046	2046	244.3
**#1233**	DCB	PR	1221	1977	247.4
**#5122**	DCB	PR	1932	1932	256.3

Supplementary Table 2

Supplementary Table 2 Detailed patient clinical data. Durable Clinical Benefit (DCB) based on Progression-free survival (PFS) > 6 months and clinical outcome determined from Best RECIST 1.1 criteria during therapy. [1]Patient only scanned at baseline. PFS and OS data updated as per March 10th 2020. Source data are provided as a Source Data file.
