# Supplementary material for: Neoantigen-specific CD8 T cell responses in the peripheral blood following PD-L1 blockade might predict therapy outcome in metastatic urothelial carcinoma
Source: Nat Commun. 2022 Apr 11;13:1935. doi: 10.1038/s41467-022-29342-0 (PMC9001725; doi:10.1038/s41467-022-29342-0)
Supplement: Supplementary file 3 — Reporting Summary [file 41467_2022_29342_MOESM3_ESM.pdf]

nature research

Corresponding author(s): Sine Reker Hadrup

Last updated by author(s): Dec 21st, 2021

Reporting Summary

Nature Research wishes to improve the reproducibility of the work that we publish. This form provides structure for consistency and transparency in reporting. For further information on Nature Research policies, see Authors & Referees and the Editorial Policy Checklist.

Statistics

For all statistical analyses, confirm that the following items are present in the figure legend, table legend, main text, or Methods section.

n/a	Confirmed	
☐	☒	The exact sample size (*n*) for each experimental group/condition, given as a discrete number and unit of measurement
☒	☐	A statement on whether measurements were taken from distinct samples or whether the same sample was measured repeatedly
☐	☒	The statistical test(s) used AND whether they are one- or two-sided<br>*Only common tests should be described solely by name; describe more complex techniques in the Methods section.*
☒	☐	A description of all covariates tested
☐	☒	A description of any assumptions or corrections, such as tests of normality and adjustment for multiple comparisons
☐	☒	A full description of the statistical parameters including central tendency (e.g. means) or other basic estimates (e.g. regression coefficient) AND variation (e.g. standard deviation) or associated estimates of uncertainty (e.g. confidence intervals)
☐	☒	For null hypothesis testing, the test statistic (e.g. *F*, *t*, *r*) with confidence intervals, effect sizes, degrees of freedom and *P* value noted<br>*Give P values as exact values whenever suitable.*
☒	☐	For Bayesian analysis, information on the choice of priors and Markov chain Monte Carlo settings
☒	☐	For hierarchical and complex designs, identification of the appropriate level for tests and full reporting of outcomes
☒	☐	Estimates of effect sizes (e.g. Cohen's *d*, Pearson's *r*), indicating how they were calculated

Our web collection on statistics for biologists contains articles on many of the points above.

Software and code

Policy information about availability of computer code

Data collection	Neopeptides were predicted using the MuPeXI pipeline, where affinity to MHC class I molecules was predicted using NetMHCpan v4.0. Prediction was conducted individually for all patients in cohort.
Data analysis	Flow cytometry data was analyzed in FlowJo, version 10.1.<br>Sequencing data from the DNA barcode-based screening was analyzed using Barracoda software version 1 (http://www.cbs.dtu.dk/services/barracoda).<br>All statistical tests were calculated in R analysis packages. The Mann Whitney Wilcoxon, Kruskal Wallis and Dunn's tests were applied as the statistic tests and R was used for the analyses.<br>Heatmaps were developed using ComplexHeatmap from Bioconductor. RNA-seq data were evaluated using Kallisto (version 0.42.1), where output was used as input in DeSeq2 (version 1.26.0) for differential expression analysis in R version 3.6.1. GO enrichment analysis was developed using R version 4.1.1 with the built in packages; enrichplot version 1.13.2 and clusterProfier version 4.0.5. Cell populations abundancy was estimated from bulk RNA sequencing data using Microenvironment Cell Populations-counter (MCP-counter). The expression matrix obtained from Kallisto was fed as input to ebecht/MCPcounter from GitHub in R version 4.0.2.<br><br>Allele copy number, purity and ploidity were found using Sequenza version 3.0. As input, bam files from normal and tumor were given to Sequenza-utils version 3.0 bam2seqz with CRCh38 as reference followed by Sequenza seqz_binding. To run the Sequenza copynumber call with CRCh38, the R packages copynumber with minor modifications from Shixiang/copynumber was applied. Sequenza results were generated with the Sequenza packages in R version (3.6.1) and copynumber information from Sequenza were merged with mutations file from Mutect2 and used as input to PyClone. To locate clonal mutations, PyClone version (0.13.0) was applied with the best estimated cellularity given from Sequenza, and max cluster of 10 and minimum size of 0 to yield all possible mutations. Afterwards, clonal mutations were filtered with a cluster size of minimum 5 and cellularity of minimum 90.

For manuscripts utilizing custom algorithms or software that are central to the research but not yet described in published literature, software must be made available to editors/reviewers. We strongly encourage code deposition in a community repository (e.g. GitHub). See the Nature Research guidelines for submitting code & software for further information.

Data

Policy information about availability of data

All manuscripts must include a data availability statement. This statement should provide the following information, where applicable:

- Accession codes, unique identifiers, or web links for publicly available datasets
- A list of figures that have associated raw data
- A description of any restrictions on data availability

All WES and RNAseq data is available upon application at dbGaP at https://www.ncbi.nlm.nih.gov/projects/gap/cgi-bin/study.cgi?study_id=phs001743.v1.p1.
GRCh38 reference genome is available at https://www.ncbi.nlm.nih.gov/assembly/GCF_000001405.39.
The source data presented in figures are provided as Source Data file. All other relevant data are available from the authors upon request.

Field-specific reporting

Please select the one below that is the best fit for your research. If you are not sure, read the appropriate sections before making your selection.

☒ Life sciences ☐ Behavioural & social sciences ☐ Ecological, evolutionary & environmental sciences

For a reference copy of the document with all sections, see nature.com/documents/nr-reporting-summary-flat.pdf

Life sciences study design

All studies must disclose on these points even when the disclosure is negative.

Sample size	The number of patients was chosen based on availability of patient material and sufficient sequencing quality. HLA-matching healthy donor samples were included concurrently to as large extent as possible, 1-3 donors per screening.
Data exclusions	No data excluded from analysis.
Replication	All patients were screened once with the patient-specific pMHC library. Detected T cell responses were evaluated by tetramer-pMHC staining to the extent possible (included in Supplementary Information), but not all detected T cell responses could be evaluated due to limited PBMC sample availability. Of 65 evaluated pMHC tetramers, 50 responses were verified, 9 borderline (combined 90%) and 6 could not be validated with this methodology.<br><br>As technical validation, in addition to the measures taken during original development of the technique (Bentzen et al., Nat Biot, 2016), applicable healthy donor samples were screened based on HLA-overlap with patient samples, and healthy donors samples were screened up to five times with different patient pMHC libraries. Results are shown in Supplementary information.
Randomization	This is a correlative study to assess T cell reactivity in relation to therapeutic outcome. Randomization is not relevant to our study. All patients were screened with patient-specific neopeptideMHC library.
Blinding	Blinding was not feasible as all patients were screened with patient-specific neopeptideMHC libraries.

Behavioural & social sciences study design

All studies must disclose on these points even when the disclosure is negative.

Study description	*Briefly describe the study type including whether data are quantitative, qualitative, or mixed-methods (e.g. qualitative cross-sectional, quantitative experimental, mixed-methods case study).*
Research sample	*State the research sample (e.g. Harvard university undergraduates, villagers in rural India) and provide relevant demographic information (e.g. age, sex) and indicate whether the sample is representative. Provide a rationale for the study sample chosen. For studies involving existing datasets, please describe the dataset and source.*
Sampling strategy	*Describe the sampling procedure (e.g. random, snowball, stratified, convenience). Describe the statistical methods that were used to predetermine sample size OR if no sample-size calculation was performed, describe how sample sizes were chosen and provide a rationale for why these sample sizes are sufficient. For qualitative data, please indicate whether data saturation was considered, and what criteria were used to decide that no further sampling was needed.*
Data collection	*Provide details about the data collection procedure, including the instruments or devices used to record the data (e.g. pen and paper, computer, eye tracker, video or audio equipment) whether anyone was present besides the participant(s) and the researcher, and whether the researcher was blind to experimental condition and/or the study hypothesis during data collection.*
Timing	*Indicate the start and stop dates of data collection. If there is a gap between collection periods, state the dates for each sample cohort.*
Data exclusions	*If no data were excluded from the analyses, state so OR if data were excluded, provide the exact number of exclusions and the rationale behind them, indicating whether exclusion criteria were pre-established.*

Non-participation	*State how many participants dropped out/declined participation and the reason(s) given OR provide response rate OR state that no participants dropped out/declined participation.*
Randomization	*If participants were not allocated into experimental groups, state so OR describe how participants were allocated to groups, and if allocation was not random, describe how covariates were controlled.*

Ecological, evolutionary & environmental sciences study design

All studies must disclose on these points even when the disclosure is negative.

Study description	*Briefly describe the study. For quantitative data include treatment factors and interactions, design structure (e.g. factorial, nested, hierarchical), nature and number of experimental units and replicates.*
Research sample	*Describe the research sample (e.g. a group of tagged Passer domesticus, all Stenocereus thurberi within Organ Pipe Cactus National Monument), and provide a rationale for the sample choice. When relevant, describe the organism taxa, source, sex, age range and any manipulations. State what population the sample is meant to represent when applicable. For studies involving existing datasets, describe the data and its source.*
Sampling strategy	*Note the sampling procedure. Describe the statistical methods that were used to predetermine sample size OR if no sample-size calculation was performed, describe how sample sizes were chosen and provide a rationale for why these sample sizes are sufficient.*
Data collection	*Describe the data collection procedure, including who recorded the data and how.*
Timing and spatial scale	*Indicate the start and stop dates of data collection, noting the frequency and periodicity of sampling and providing a rationale for these choices. If there is a gap between collection periods, state the dates for each sample cohort. Specify the spatial scale from which the data are taken*
Data exclusions	*If no data were excluded from the analyses, state so OR if data were excluded, describe the exclusions and the rationale behind them, indicating whether exclusion criteria were pre-established.*
Reproducibility	*Describe the measures taken to verify the reproducibility of experimental findings. For each experiment, note whether any attempts to repeat the experiment failed OR state that all attempts to repeat the experiment were successful.*
Randomization	*Describe how samples/organisms/participants were allocated into groups. If allocation was not random, describe how covariates were controlled. If this is not relevant to your study, explain why.*
Blinding	*Describe the extent of blinding used during data acquisition and analysis. If blinding was not possible, describe why OR explain why blinding was not relevant to your study.*

Did the study involve field work? ☐ Yes ☐ No

Field work, collection and transport

Field conditions	*Describe the study conditions for field work, providing relevant parameters (e.g. temperature, rainfall).*
Location	*State the location of the sampling or experiment, providing relevant parameters (e.g. latitude and longitude, elevation, water depth).*
Access and import/export	*Describe the efforts you have made to access habitats and to collect and import/export your samples in a responsible manner and in compliance with local, national and international laws, noting any permits that were obtained (give the name of the issuing authority, the date of issue, and any identifying information).*
Disturbance	*Describe any disturbance caused by the study and how it was minimized.*

Reporting for specific materials, systems and methods

We require information from authors about some types of materials, experimental systems and methods used in many studies. Here, indicate whether each material, system or method listed is relevant to your study. If you are not sure if a list item applies to your research, read the appropriate section before selecting a response.

Materials & experimental systems

n/a	Involved in the study
☐	☒ Antibodies
☒	☐ Eukaryotic cell lines
☒	☐ Palaeontology
☒	☐ Animals and other organisms
☐	☒ Human research participants
☐	☒ Clinical data

Methods

n/a	Involved in the study
☒	☐ ChIP-seq
☐	☒ Flow cytometry
☒	☐ MRI-based neuroimaging

Antibodies

Antibodies used

CD8-BV480 (BD, cat. #566121, clone RPA-T8, 2µl), dump channel antibodies (CD4-FITC (BD, cat. #345768, 1.25µl), CD14-FITC (BD, cat. #345784, 3.125µl), CD19-FITC (BD, cat. #345776, 6.25µl), CD40-FITC (Serotech, cat. #MCA1590F, 2.5µl), and CD16-FITC (BD, cat. #335035, 1.56µl)) and a dead cell marker (LIVE/DEAD Fixable Near-IR; Invitrogen, cat. #L10119, 0.1µl).

CD3-BV786 (BD, cat. #563799, clone SK7, 5µl), CD4-BV650 (BD, cat. #563876, 2.5µl), CD8-BV480 (BD, cat. #566121, clone RPA-T8, 2µl), Ki67-BUV395 (BD, cat. #564071, clone B56, 2.5µl), 4-1BB-BUV737 (BD, cat. #741867, clone 4B4-1, 2.5µl), PD1-BV421 (BioLegend, cat. #329920, clone EH12.2H7, 3µl), CD27-BV605 (BioLegend, cat. #302829, clone O323, 2.5µl), CD45RA-BV711 (BD, cat. #563733, clone HI100, 2.5µl), CCR7-FITC (BioLegend, cat. #353215, clone G043H7, 5µl), Eomes-PerCP-eFlour710 (eBioscience, Thermo Fisher Scientific cat. #46-4877-41, clone WD1928, 2.5µl), CD39-PE-CF594 (BD, cat. #563678, clone Tu66, 2.5µl), CD57-PECy7 (BioLegend, cat. #393309, clone QA17A04, 2.5µl), and GranzymeB-AlexaFlour700 (BioLegend, cat. #372221, clone QA16A02, 1.25µl), and a dead cell marker (LIVE/DEAD Fixable Near-IR; Invitrogen, cat. #L10119, 0.1µl).

Validation

Validation generally provided and ensured by manufacturer (BD, BioLegend, Serotech and Invitrogen). Following procurement from manufacturer, each antibody was tested and titrated using human PBMCs to ensure correct performance in the relevant setting, and further validated by staining of resting human PBMCs and in vitro-expanded PBMCs to ensure target expression levels and adequate antibody concentrations.

For 4-1BB-BUV737 (BD), manufactorer validation statements were stated: "The production process underwent stringent testing and validation to assure that it generates a high-quality conjugate with consistent performance and specific binding activity." For LIVE/DEAD Fixable Near-IR (Invitrogen): "This kit has been optimized and validated for use with a red laser flow cytometer." No other antibody-specific statements could be found.

Serotech states that "In house manufactured antibodies that have 'flow cytometry' indicated on the datasheet have been assessed to ensure application suitability. This includes testing the antibody for performance in flow cytometry using a relevant cell line, or primary cells, known to express the marker under appropriate conditions. They also have to meet internal criteria including batch-to-batch consistency for dilution, percentage of positive cells, and shift."

Eukaryotic cell lines

Policy information about cell lines

Cell line source(s)

State the source of each cell line used.

Authentication

Describe the authentication procedures for each cell line used OR declare that none of the cell lines used were authenticated.

Mycoplasma contamination

Confirm that all cell lines tested negative for mycoplasma contamination OR describe the results of the testing for mycoplasma contamination OR declare that the cell lines were not tested for mycoplasma contamination.

Commonly misidentified lines
(See ICLAC register)

Name any commonly misidentified cell lines used in the study and provide a rationale for their use.

Palaeontology

Specimen provenance

Provide provenance information for specimens and describe permits that were obtained for the work (including the name of the issuing authority, the date of issue, and any identifying information).

Specimen deposition

Indicate where the specimens have been deposited to permit free access by other researchers.

Dating methods

If new dates are provided, describe how they were obtained (e.g. collection, storage, sample pretreatment and measurement), where they were obtained (i.e. lab name), the calibration program and the protocol for quality assurance OR state that no new dates are provided.

☐ Tick this box to confirm that the raw and calibrated dates are available in the paper or in Supplementary Information.

Animals and other organisms

Policy information about studies involving animals; ARRIVE guidelines recommended for reporting animal research

Laboratory animals	*For laboratory animals, report species, strain, sex and age OR state that the study did not involve laboratory animals.*
Wild animals	*Provide details on animals observed in or captured in the field; report species, sex and age where possible. Describe how animals were caught and transported and what happened to captive animals after the study (if killed, explain why and describe method; if released, say where and when) OR state that the study did not involve wild animals.*
Field-collected samples	*For laboratory work with field-collected samples, describe all relevant parameters such as housing, maintenance, temperature, photoperiod and end-of-experiment protocol OR state that the study did not involve samples collected from the field.*
Ethics oversight	*Identify the organization(s) that approved or provided guidance on the study protocol, OR state that no ethical approval or guidance was required and explain why not.*

Note that full information on the approval of the study protocol must also be provided in the manuscript.

Human research participants

Policy information about studies involving human research participants

Population characteristics	Patients had mUC and were treated with atezolizumab 1200mg intravenously (IV) every 21 days at Memorial Sloan Kettering Cancer Center as part of the IMVigor210 trial (Rosenberg et al. 2016). 24 patients with varying clinical outcome were including in the cohort (n = 13 progressive disease, n = 4 stable disease, n = 2 partial response and n = 5 complete response patients according to Best RECIST 1.1).<br>A description of covariate data is previously published (Snyder et al. 2017, https://pubmed.ncbi.nlm.nih.gov/28552987/), ref 19 in manuscript file.
Recruitment	Patient samples were included from previous study (Snyder et al., 2017) and based on material availability and sequencing quality.
Ethics oversight	The relevant institutional ethics committees approved the study and informed written consent was obtained in accordance with the Declaration of Helsinki.<br>The Institution Review Board of Memorial Sloan Kettering Cancer Center approved a biospecimen protocol permitting tissue and blood collection, sequencing, and correlative studies for patients on this trial. All patients provided written informed consent to this protocol. All patients provided written informed consent to both the IMvigor 210 trial and an Institution Review Board-approved biospecimen protocol permitting tissue and blood collection, sequencing, and correlative studies. We have included documentation to support this claim. Of note, all clinical and genomic data are publicly available for download by authorized investigators on dbGaP at http://view.ncbi.nlm.nih.gov/dbgap-controlled.<br>HD samples were collected by approval of the local Scientific Ethics Committee for the Capital Region of Denmark, with donor written informed consent obtained according to the Declaration of Helsinki. HD blood samples were obtained from the blood bank at Rigshospitalet, Copenhagen, Denmark. All samples were obtained anonymously.

Note that full information on the approval of the study protocol must also be provided in the manuscript.

Clinical data

Policy information about clinical studies
All manuscripts should comply with the ICMJE guidelines for publication of clinical research and a completed CONSORT checklist must be included with all submissions.

Clinical trial registration	ClinicalTrials.gov, number NCT02108652.
Study protocol	Patient samples were collected from at Memorial Sloan Kettering Cancer Center as part of the IMVigor210 trial.
Data collection	Patients had mUC and were treated with atezolizumab 1200mg intravenously (IV) every 21 days at Memorial Sloan Kettering Cancer Center as part of the IMVigor210 trial (Rosenberg et al. 2016). Cross sectional imaging was performed every 9 weeks for the first 12 months following cycle 1 day 1 followed by every 12 weeks thereafter.
Outcomes	The best overall clinical treatment response was determined by radiologic assessment of response, using RECIST version 1.1. In patients in whom clinical progression was determined based on symptoms and decline and functional status, this determination superseded radiologic categorization.

ChIP-seq

Data deposition

- ☐ Confirm that both raw and final processed data have been deposited in a public database such as GEO.
- ☐ Confirm that you have deposited or provided access to graph files (e.g. BED files) for the called peaks.

Data access links
May remain private before publication.

For "Initial submission" or "Revised version" documents, provide reviewer access links. For your "Final submission" document, provide a link to the deposited data.

Files in database submission

Provide a list of all files available in the database submission.

Genome browser session
(e.g. UCSC)

Provide a link to an anonymized genome browser session for "Initial submission" and "Revised version" documents only, to enable peer review. Write "no longer applicable" for "Final submission" documents.

Methodology

Replicates	*Describe the experimental replicates, specifying number, type and replicate agreement.*
Sequencing depth	*Describe the sequencing depth for each experiment, providing the total number of reads, uniquely mapped reads, length of reads and whether they were paired- or single-end.*
Antibodies	*Describe the antibodies used for the ChIP-seq experiments; as applicable, provide supplier name, catalog number, clone name, and lot number.*
Peak calling parameters	*Specify the command line program and parameters used for read mapping and peak calling, including the ChIP, control and index files used.*
Data quality	*Describe the methods used to ensure data quality in full detail, including how many peaks are at FDR 5% and above 5-fold enrichment.*
Software	*Describe the software used to collect and analyze the ChIP-seq data. For custom code that has been deposited into a community repository, provide accession details.*

Flow Cytometry

Plots

Confirm that:

☒ The axis labels state the marker and fluorochrome used (e.g. CD4-FITC).

☒ The axis scales are clearly visible. Include numbers along axes only for bottom left plot of group (a 'group' is an analysis of identical markers).

☒ All plots are contour plots with outliers or pseudocolor plots.

☒ A numerical value for number of cells or percentage (with statistics) is provided.

Methodology

Sample preparation	Patient formalin-fixed paraffin-embedded (FFPE) tumor and PBMC samples were obtained and prepared as previously described (Snyder et al. 2017). Blood samples were drawn from patients prior to IV infusion on the day of treatment, pre-treatment and during treatment, and PBMCs were isolated and cryopreserved at -150°C in Human Serum Albumin (HSA)/10% DMSO until analysis.<br>Healthy donor blood samples were obtained from the blood bank at Rigshospitalet, Copenhagen, Denmark. All samples were obtained anonymously. PBMCs from HDs were obtained from whole blood by density centrifugation on Lymphoprep in Leucosep tubes and cryopreserved at -150°C in FCS + 10% DMSO.
Instrument	Cells were sorted on a BD FACSMelody, BD FACSAriaFusion or acquired on a BD LSR Fortessa.
Software	FlowJo software was used to gate and sort the population of interest
Cell population abundance	For every T cell population sorted the sorted cell fraction represented 0.5-25% of the total population.
Gating strategy	For gating on BD FACSMelody and BD LSR Fortessa: Lymphocytes were defined within a FSC/SSC plot. Among these we gated on single (FSC-A/FSC-H), live (NIR negative), CD8 positive (BV480) and 'dump' (CD4, 14, 16, 19, and 40) (FITC) negative cells and sorted/acquired all mulimer/tetramer-positive (PE or tetramer-streptaviding) cells.<br>For gating on BD FACSAriaFusion: Lymphocytes, single, live cells gated as above. CD3 positive (BV786), CD8 positive (BV480) and CD4 (BV650) negative cells and sorted all multimer-positive (PE or APC) cells.

☒ Tick this box to confirm that a figure exemplifying the gating strategy is provided in the Supplementary Information.

Magnetic resonance imaging

Experimental design

Design type	*Indicate task or resting state; event-related or block design.*

Design specifications	*Specify the number of blocks, trials or experimental units per session and/or subject, and specify the length of each trial or block (if trials are blocked) and interval between trials.*
Behavioral performance measures	*State number and/or type of variables recorded (e.g. correct button press, response time) and what statistics were used to establish that the subjects were performing the task as expected (e.g. mean, range, and/or standard deviation across subjects).*

Acquisition

Imaging type(s)	*Specify: functional, structural, diffusion, perfusion.*
Field strength	*Specify in Tesla*
Sequence & imaging parameters	*Specify the pulse sequence type (gradient echo, spin echo, etc.), imaging type (EPI, spiral, etc.), field of view, matrix size, slice thickness, orientation and TE/TR/flip angle.*
Area of acquisition	*State whether a whole brain scan was used OR define the area of acquisition, describing how the region was determined.*

Diffusion MRI ☐ Used ☐ Not used

Preprocessing

Preprocessing software	*Provide detail on software version and revision number and on specific parameters (model/functions, brain extraction, segmentation, smoothing kernel size, etc.).*
Normalization	*If data were normalized/standardized, describe the approach(es): specify linear or non-linear and define image types used for transformation OR indicate that data were not normalized and explain rationale for lack of normalization.*
Normalization template	*Describe the template used for normalization/transformation, specifying subject space or group standardized space (e.g. original Talairach, MNI305, ICBM152) OR indicate that the data were not normalized.*
Noise and artifact removal	*Describe your procedure(s) for artifact and structured noise removal, specifying motion parameters, tissue signals and physiological signals (heart rate, respiration).*
Volume censoring	*Define your software and/or method and criteria for volume censoring, and state the extent of such censoring.*

Statistical modeling & inference

Model type and settings	*Specify type (mass univariate, multivariate, RSA, predictive, etc.) and describe essential details of the model at the first and second levels (e.g. fixed, random or mixed effects; drift or auto-correlation).*
Effect(s) tested	*Define precise effect in terms of the task or stimulus conditions instead of psychological concepts and indicate whether ANOVA or factorial designs were used.*

Specify type of analysis: ☐ Whole brain ☐ ROI-based ☐ Both

Statistic type for inference<br>(See Eklund et al. 2016)	*Specify voxel-wise or cluster-wise and report all relevant parameters for cluster-wise methods.*
Correction	*Describe the type of correction and how it is obtained for multiple comparisons (e.g. FWE, FDR, permutation or Monte Carlo).*

Models & analysis

n/a	Involved in the study
☐	☐ Functional and/or effective connectivity
☐	☐ Graph analysis
☐	☐ Multivariate modeling or predictive analysis

Functional and/or effective connectivity	*Report the measures of dependence used and the model details (e.g. Pearson correlation, partial correlation, mutual information).*
Graph analysis	*Report the dependent variable and connectivity measure, specifying weighted graph or binarized graph, subject- or group-level, and the global and/or node summaries used (e.g. clustering coefficient, efficiency, etc.).*
Multivariate modeling and predictive analysis	*Specify independent variables, features extraction and dimension reduction, model, training and evaluation metrics.*
